# Information-Theoretic Perspectives on Chemical Problems: Recent Developments and Applications

**DOI:** 10.3390/e28030277

**Published:** 2026-03-01

**Authors:** Arpita Poddar, Pratim Kumar Chattaraj

**Affiliations:** 1 Department of Chemistry, Indian Institute of Technology Kharagpur, Kharagpur 721302, India; 2 Department of Chemistry, Birla Institute of Technology Mesra, Ranchi 835215, India

**Keywords:** information-theoretic approach, density functional theory, conceptual DFT, descriptors

## Abstract

Information-theoretic approach (ITA) has emerged as a powerful density-based framework for interpreting molecular structure, stability, and reactivity within density functional theory (DFT). By treating the electron density as a probability distribution, information-theoretic (IT) descriptors provide physically transparent measures of electron delocalization, localization, and density reorganization, offering an alternative to traditional orbital-based interpretations. This review presents a focused account of the theoretical foundations and chemical significance of IT descriptors and highlights their growing role in density-based chemical analysis. Selected applications are discussed to illustrate how these measures successfully rationalize molecular stability, bonding patterns, reactivity trends, and structure–property relationships across diverse chemical systems. The interplay between IT descriptors and conceptual DFT quantities is also examined, emphasizing their complementary nature in chemical reactivity studies. Overall, this review underscores the versatility and predictive capability of information-theoretic functionals of the electron density and their potential to advance a unified, orbital-free framework for understanding chemical behavior.

## 1. Introduction

DFT [1] has become one of the most successful and widely used frameworks for investigating the electronic structure of atoms, molecules, and condensed-phase systems. Its conceptual foundation rests on the Hohenberg–Kohn theorems [1,2], which establish the ground-state electron density as the fundamental variable containing complete information about a many-electron system. This density-based formulation bypasses the explicit treatment of the many-electron wave function, offering both conceptual clarity and computational efficiency. In recent decades, DFT has not only matured as a practical electronic structure method but has also evolved into a powerful platform for developing chemical reactivity theories.

An important development in this direction is conceptual density functional theory (CDFT) [3], where long-standing chemical concepts, such as electronegativity [4,5,6,7], hardness [2,8], softness [9,10], and electrophilicity [11], are rigorously defined in terms of energy and density derivatives. By treating chemical reactivity as the system’s response to changes in electron number or external potential, CDFT provides a quantitative and internally consistent framework for rationalizing molecular stability, selectivity, and reactivity patterns. These descriptors have been successfully applied to a broad range of chemical problems, including structure–activity relationships, toxicity assessment, and reaction mechanism analysis [12,13,14]. Nevertheless, many CDFT descriptors rely on finite-difference energy derivatives, orbital energies, or population analysis schemes, which introduce methodological ambiguities and limit transferability across different computational approaches.

In parallel with these developments, there has been a growing effort to formulate chemical reactivity directly in terms of the electron density and its associated functionals, without recourse to orbitals or response derivatives. This endeavor naturally connects DFT with information theory, a mathematical framework originally developed to quantify uncertainty and information content in probability distributions. Since the electron density can be interpreted as a continuous probability distribution, information-theoretic concepts provide a physically meaningful and formally consistent language for analyzing electronic structure. This line of research is now broadly known as the information-theoretic approach (ITA) [15,16,17] within DFT. The extension of information-theoretic descriptors toward the direct description of chemical reactivity has led to the development of density functional reactivity theory (DFRT) [2,15,18,19], in which molecular stability and reactivity are characterized using density-based information measures rather than orbital concepts.

The ITA introduces a class of simple yet powerful density functionals such as Shannon entropy [20], Fisher information [21], Ghosh–Berkowitz–Parr entropy [22], Rényi and Tsallis entropies [23,24,25], Onicescu information energy [26], and information gain [27,28] that quantify complementary aspects of electronic organization. These measures characterize electron delocalization, localization, kinetic energy distribution, and density reorganization relative to reference states. Importantly, they are explicit functionals of the electron density and its derivatives, making them conceptually aligned with the foundational principles of DFT. Applications over the last two decades have demonstrated that IT descriptors can successfully rationalize chemical bonding, aromaticity, steric effects, molecular stability, reactivity trends, and even energetic components such as exchange–correlation energies [29,30,31,32,33,34,35,36,37,38,39,40,41,42,43,44,45,46,47,48,49,50,51,52].

Beyond individual descriptors, information theory offers a unified framework for analyzing molecular systems through entropic measures of information distance, enabling direct comparisons between molecular electron densities and their promolecular or fragment-based reference distributions. This perspective allows the extraction of entropic characteristics from both continuous and discretized electron probability distributions, as encountered in atoms-in-molecules (AIM) analyses. Moreover, the ITA facilitates the construction of density-based indices related to bonding and multiplicity and supports a thermodynamic-like interpretation of electronic structure by integrating entropy-based principles with the energetic variational foundation of DFT. Such integration significantly enriches the physical interpretation of molecular electron distributions.

Recent advances further suggest that IT quantities are not independent but intrinsically correlated, as dictated by the first Hohenberg–Kohn theorem. This realization has motivated the emergence of information functional theory (IFT) [52], where multiple IT descriptors are employed collectively to describe total energies, electronic properties, and chemical behavior. Beyond their theoretical appeal, IT descriptors are computationally affordable and readily transferable, making them particularly attractive for large-scale applications such as Quantitative Structure–Activity/Property Relationship (QSAR/QSPR) modeling, toxicity prediction, and materials screening. When combined with data-driven approaches, they offer a robust bridge between fundamental electronic structure theory and predictive chemical modeling.

In contrast to earlier reviews that primarily focus on the formal definition of information-theoretic measures or their application to chemical bonding and atomic structure, the present work emphasizes the recent evolution of ITA toward a genuine reactivity theory. Specifically, we highlight the emergence of DFRT and IFT as integrative frameworks, discuss newly explored application areas such as QSAR/QSPR modeling and toxicity prediction, and outline how IT descriptors are increasingly combined with data-driven methods. By synthesizing these developments, this review aims to provide a forward-looking perspective on how information theory is reshaping density-based chemical reactivity concepts.

Importantly, the diverse applications discussed throughout this review are not independent case studies, but rather different manifestations of a single information-theoretic principle: chemical structure, stability, and reactivity arise from how the electron density is organized and redistributed in space. From this perspective, entropy measures, information distances, and kinetic-energy-based descriptors provide complementary projections of the same underlying density-based information, offering a unified alternative to traditional orbital-based interpretations.

Therefore, this review focuses on the theoretical foundations, chemical interpretation, and recent applications of the information-theoretic approach within DFT. Rather than providing an exhaustive survey, we aim to synthesize key concepts, highlight emerging trends, and clarify how IT descriptors enrich our understanding of molecular structure and reactivity. By emphasizing their physical meaning and chemical relevance, this review seeks to position ITA as a unifying and forward-looking framework for density-based chemical theory.

## 2. Theoretical Foundations of Information Theory in DFT

Information theory provides a rigorous mathematical framework for quantifying uncertainty, localization, and redistribution in probability distributions. Since the electron density is a normalized, continuous probability distribution that uniquely determines all ground-state properties of a system, information-theoretic measures can be naturally embedded within density functional theory (DFT). The information-theoretic approach (ITA) thus offers a complementary, density-based language for understanding electronic structure, bonding, and reactivity.

### 2.1. Electron Density as a Probability Distribution

In ITA, the total electron density *ρ*(r) is treated as a probability distribution function normalized to the number of electrons:

$$\int \rho \left(r\right)dr=N.$$

This probabilistic interpretation allows the direct application of entropy- and information-based measures originally developed in information theory. Unlike abstract probability distributions, however, the electron density carries complete physical information about the ground state, as guaranteed by the Hohenberg–Kohn theorems [1,2]. Consequently, information-theoretic quantities derived from *ρ*(r) are not independent but encode overlapping aspects of the same underlying electronic structure.

### 2.2. Shannon Entropy and Electronic Delocalization

The Shannon entropy [20] is the most widely used information measure in ITA and is defined as

$$S_{S}=-\int \rho \left(r\right)\ln\rho \left(r\right)dr.\;$$

It quantifies the degree of spatial delocalization of the electron density. Larger Shannon entropy values correspond to more diffuse, uniformly distributed densities, while smaller values indicate localized electronic structures. The local Shannon entropy density provides spatially resolved insight into regions of enhanced delocalization and has been successfully used to analyze molecular stability, aromaticity, and conformational preferences.

### 2.3. Fisher Information and Localization

Complementary to Shannon entropy [20], Fisher information [21] measures the sharpness or localization of the electron density:

$$I_{F\;\;}=\int \frac{{\left|\nabla \rho (r)\right|}^{2}}{\rho (r)}dr.$$

High Fisher information indicates strong density gradients and localized electrons, while low values correspond to smoother, more delocalized distributions. Fisher information vanishes for homogeneous electron densities and is directly related to the Weizsäcker kinetic energy, being exactly eight times its value. An alternative, Laplacian-based formulation of Fisher information exists, that is globally equivalent but locally distinct, offering greater sensitivity to bonding features and steric effects.

### 2.4. GBP Entropy and the Kinetic Energy Connection

The Ghosh–Berkowitz–Parr (GBP) entropy [22] introduces kinetic energy effects into ITA through a phase-space-inspired formulation:

$$S_{GBP}=\;\int \frac{3}{2}k\rho (r)\left[c+\;\ln\frac{t(r;\rho )}{t_{TF}(r;\rho )}\right]dr,$$

where t(r;ρ) is the kinetic energy density and *t*_TF_ (*r*;*ρ*) is the Thomas–Fermi kinetic energy density. Unlike Shannon entropy, which reflects spatial delocalization, GBP entropy captures both real-space and momentum-space characteristics of the electrons. It is particularly sensitive to changes in bonding, steric congestion, and kinetic energy redistribution, making it a powerful descriptor for chemical interactions and reactivity trends.

### 2.5. Information Gain and Reference Densities

Information gain (*I_G_*) [26,27,28], also known as the Kullback–Leibler divergence, measures the information difference between a target density $\rho \left(r\right)$ and a reference density $\rho_{0}(r)$:

$$I_{G\;}=\;\int \rho \left(r\right)\ln\frac{\rho (r)}{\rho_{0}(r)}dr$$

The reference density can represent isolated atoms, a reactant state, or an alternative molecular conformation. Information gain therefore quantifies electronic redistribution during chemical processes, such as bond formation, conformational changes, or reactions. Exact identities relate *I_G_* to Laplacian- and gradient-based contributions, revealing its deep connection to steric, electrostatic, and uniformity effects in molecular systems.

*I_G_* can also be expressed through further calculations as

$$I_{G}=-\frac{1}{4\pi }\iint \frac{g_{1}\left(r^{\prime }\right)}{\left|r-r^{\prime }\right|}drdr^{\prime }-\frac{1}{4\pi }\iint \frac{g_{2}\left(r^{\prime }\right)}{\left|r-r^{\prime }\right|}drdr^{\prime }-\frac{1}{4\pi }\iint \frac{g_{3}\left(r^{\prime }\right)}{\left|r-r^{\prime }\right|}drdr^{\prime }$$

The functions *g*_1_, *g*_2_, and *g*_3_ in this expression offer additional chemical insights and are defined as follows [27]:

$$g_{1}\left(r^{\prime }\right)={𝛻}^{2}\rho \left(r^{\prime }\right)ln\frac{\rho \left(r^{\prime }\right)}{\rho_{0}\left(r^{\prime }\right)}$$

$$g_{2}\left(r^{\prime }\right)=\rho \left(r^{\prime }\right)\left[\frac{{𝛻}^{2}\rho \left(r^{\prime }\right)}{\rho \left(r^{\prime }\right)}-\frac{{𝛻}^{2}\rho_{0}\left(r^{\prime }\right)}{\rho_{0}\left(r^{\prime }\right)}\right]$$

$$g_{3}\left(r^{\prime }\right)=\rho \left(r^{\prime }\right){\left[𝛻ln\frac{\rho \left(r^{\prime }\right)}{\rho_{0}\left(r^{\prime }\right)}\right]}^{2}$$

### 2.6. Rényi, Tsallis, and Onicescu Measures

Beyond Shannon entropy, several generalized entropy measures have been introduced in ITA. Rényi entropy of order n [23,24,25] provides a tunable measure of density dispersion and reduces to Shannon entropy as n→1. Tsallis entropy generalizes the Boltzmann–Gibbs framework and is particularly useful for systems exhibiting non-extensive behavior. Closely related to both is the Onicescu information energy, which depends on integrals of powers of the electron density and offers a refined measure of density concentration. These quantities allow selective emphasis on different regions of the electron density and have been shown to correlate with stability, reactivity, and energetic trends.

The corresponding mathematical equations are as follows:

Rényi entropy of order *n* (*n* ≥0, n≠1) [23]:

$$R_{n\;}=\;\frac{1}{1-n}ln\left[\int {\rho (r)}^{n}dr\right]$$

The respective quadratic form: $-log\int \rho^{2}\left(r\right)dr$

Cubic form: $-\left(1/2\right)log\int \rho^{3}\left(r\right)dr$

The relative Rényi entropy of order *n* [24,25]:

$$R_{n}^{r}=\;\frac{1}{n-1}ln\left[\int \frac{\rho^{n}(r)}{\rho_{0}^{n-1}(r)}dr\right]$$

Quadratic form: $-log\sum \int \rho^{2}(r)/\rho_{0}\left(r\right)dr$

Cubic form: $-log\sum \int \rho^{3}(r)/\rho_{0}^{2}\left(r\right)dr$

Tsallis entropy (*T_n_*) [53] and Onicescu information energy (*E_n_*) [26]:

$$T_{n}=\;\frac{1}{n-1\;}\left[1-\;\int \rho^{n}(r)dr\right]$$

$$E_{n}=\frac{1}{n-1}\int \rho^{n}(r)dr\;\;(n\geq 2)$$

### 2.7. Local vs. Global Information-Theoretic Descriptors

Information-theoretic quantities can be defined at both global and local levels. While global descriptors characterize the overall electronic structure of a system, local densities such as local Shannon entropy or Fisher information density enable spatially resolved analyses. Furthermore, ITA can be formulated in alternative representations, including the shape function framework [40], where the electron density is normalized per electron, and atom-in-molecule partitions, enabling atomic decomposition of information measures. Rigorous analytical relationships connect these representations, ensuring that physical insights remain consistent across different formulations.

## 3. Information-Theoretic Descriptors and Chemical Concepts

A major strength of ITA within DFT lies in its ability to reinterpret traditional chemical concepts directly in terms of the electron density. Since the electron density uniquely determines the ground-state properties of a system, information-theoretic descriptors, being explicit density functionals, offer an alternative yet fully consistent language to describe stability, bonding, aromaticity, and reactivity.

### 3.1. Chemical Stability and Electronic Organization

Chemical stability is closely linked to how electrons are distributed in space. Shannon entropy provides a global measure of density delocalization, with lower entropy generally associated with more compact and energetically stable electronic arrangements. Conversely, Fisher information quantifies density localization and sharp gradients, increasing in systems with tightly bound electrons or pronounced shell structures. The complementary nature of these two descriptors reflects the balance between delocalization and localization that underlies energetic stabilization. Relative entropies, such as information gain, further quantify changes in stability by measuring how much a system’s density deviates from a reference state, making them particularly useful for tracking reactions, conformational changes, and binding processes.

### 3.2. Aromaticity and Electron Delocalization

Aromaticity, traditionally described in terms of resonance and cyclic electron delocalization, finds a natural interpretation within ITA. Aromatic systems typically exhibit enhanced Shannon entropy due to uniform electron spreading over the molecular framework, while Fisher information is reduced as sharp density variations are suppressed. Information gain and relative entropy measures have been shown to distinguish aromatic, antiaromatic, and nonaromatic systems by comparing molecular densities to suitable reference distributions. In this sense, IT descriptors provide a density-based, quantitative complement to classical aromaticity indices.

### 3.3. Hardness, Softness, and Qualitative Reactivity Trends

Although conceptual DFT defines hardness and softness through energy derivatives, IT descriptors capture related trends qualitatively through density organization. Systems with high Fisher information and low Shannon entropy tend to resist density deformation, corresponding to “harder” chemical behavior. In contrast, soft, highly polarizable systems often exhibit greater delocalization and lower density gradients, reflected in higher entropy and reduced Fisher information. Thus, IT descriptors encode reactivity tendencies without explicit reference to orbital energies or response functions.

### 3.4. Physical Meaning of Information Measures in Chemistry

Each information-theoretic quantity carries a clear chemical interpretation. Shannon entropy measures the extent of electron delocalization and spatial uniformity. Fisher information reflects localization, steric congestion, and kinetic energy-related effects. GBP entropy links density inhomogeneity to kinetic energy and bonding characteristics. Relative entropies quantify electronic reorganization during chemical processes. Together, these descriptors transform abstract information measures into physically intuitive tools for understanding molecular structure and reactivity.

## 4. Applications of ITA to Chemical Systems

Recent years have witnessed extensive applications of IT descriptors to explore a wide range of physicochemical properties in inorganic, organic, and biologically relevant systems. By employing entropy- and information-based density functionals either individually or in combination, ITA has proven capable of rationalizing energetic trends, stability, and reactivity with remarkable quantitative accuracy.

**Figure 1:**
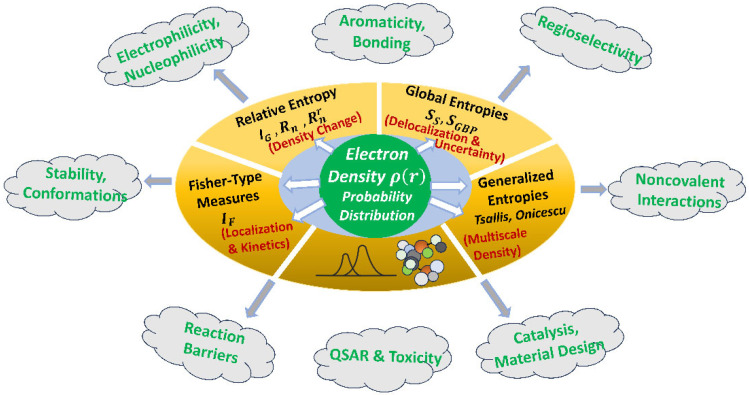
Schematic classification of information-theoretic descriptors in DFT, highlighting their mathematical forms, physical meanings, and representative chemical applications.

Conceptually, all of these studies share a common foundation: changes in stability, bonding, and reactivity are traced back to how the electron-density probability distribution reorganizes in response to structural, environmental, or external perturbations. In this sense, each application represents a different chemical projection of the same underlying information-theoretic description of electronic structure. Figure 1 depicts the schematic overview of the information-theoretic framework in DFT, classifying the main classes of information-theoretic descriptors according to their mathematical form, physical interpretation and typical applications to chemical structure, stability, and reactivity.

### 4.1. Conformational Effects and Anomeric Interactions

Cao et al. [51] investigated the anomeric effect in a series of 45 molecular systems of the general form R_1_-X-CH_2_-R_2_ (X = O, S, Se; R_1_/R_2_ = Me, F, Cl, NH_2_, OH, OMe, CH=CH_2_, C≡CH) using IT descriptors in conjunction with two energy-partitioning schemes. A moderate correlation (*R*^2^ = 0.67) was observed between the energy difference (∆*E*) and *I_G_* for the anti and gauche conformers of CH_3_CH_2_OCH_3_ and FCH_2_OF. Notably, when multiple IT descriptors, *viz*., *S_S_*, *I_F_*, *E_n_*, *S_GBP_*, *I_G_*, and *R_n_*, were combined via multilinear regression, an excellent correlation (*R*^2^ = 0.99) was achieved. These results highlight the advantage of descriptor synergy in predictive modeling. For most systems, the gauche conformer was found to be more stable than the anti-form, with electrostatic interactions identified as the dominant stabilizing factor.

### 4.2. Molecular Acidity Prediction

**Scheme 1:**
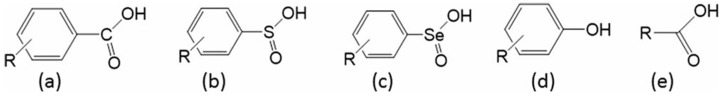
Representative acidic systems examined in this work: (**a**) substituted benzoic acids (mono- and disubstituted), (**b**) benzenesulfinic acids, (**c**) benzeneseleninic acids, (**d**) substituted phenols, and (**e**) alkyl carboxylic acids. (Reprinted from Reference [48] with permission from John Wiley and Sons. © 2017, Wiley Periodicals, Inc.).

**Figure 2:**
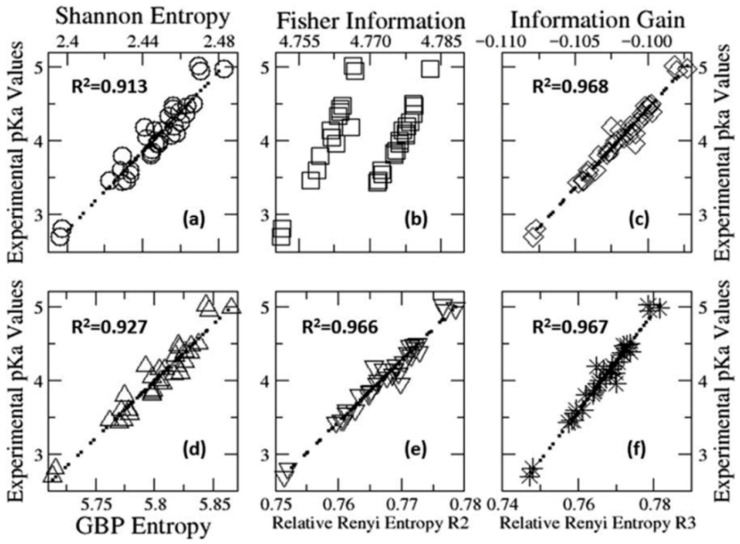
Plots correlating experimentally determined pKa values with different IT-related quantities. (**a**–**f**) represent the strong linear correlation between various IT parameters with the experimental pKa values. (Reprinted from Reference [48] with permission from John Wiley and Sons. © 2017, Wiley Periodicals, Inc.).

**Figure 3:**
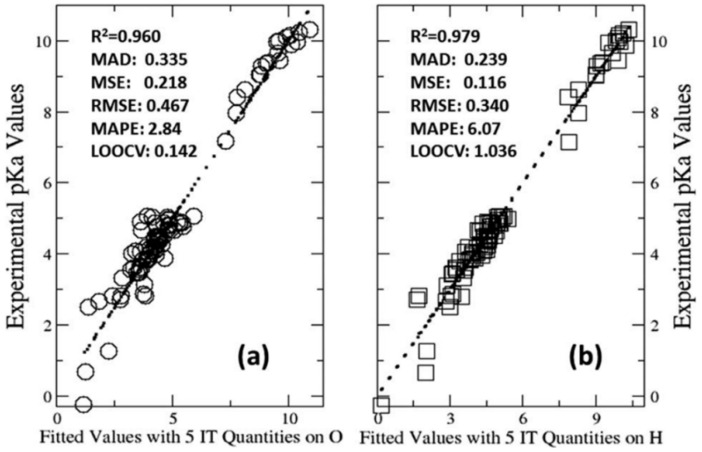
Plot comparing experimental pKa values for four series of acidic compounds, benzenesulfinic acid, benzeneseleninic acid, benzoic acid, and alkyl carboxylic acid derivatives, with pKa values fitted using five IT quantities for (**a**) the acidic oxygen atom and (**b**) the dissociating proton. (Reprinted from Reference [48] with permission from John Wiley and Sons. © 2017, Wiley Periodicals, Inc.).

The same group further extended ITA to quantify molecular acidity within the density functional reactivity theory framework [48]. Five distinct acidic series (Scheme 1) were examined using IT descriptors to correlate with experimental pKa values. As illustrated in Figure 2, strong linear relationships were obtained for singly substituted benzoic acid derivatives. Regression analyses employing five IT descriptors (*S_S_*, *S_GBP_*, *I_G_*, $R_{n}^{2}$, $R_{n}^{3}$) for the acidic oxygen atom and the dissociating proton yielded correlation coefficients of 0.96 and 0.98, respectively, for the datasets shown in Figure 3. The robustness of these models was further supported by low MAD, MSE, RMSE, and MAPE values.

Extending this perspective from local functional groups to global π-electron delocalization, IT descriptors also capture aromaticity and conformational effects as information-theoretic measures of electron sharing.

### 4.3. Aromaticity and π-Electron Delocalization

**Figure 4:**
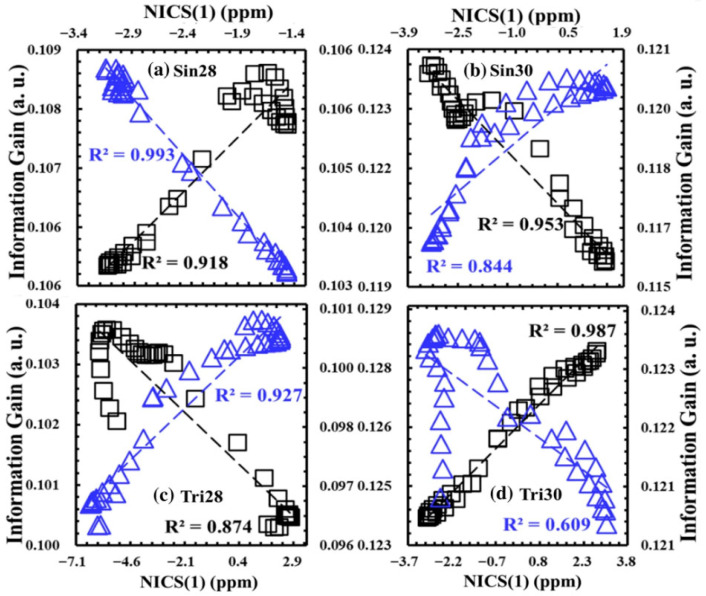
Correlation between NICS values and information gain resulting from rotational motions of the two benzene rings. (Reprinted from Reference [50] with permission from Springer Nature. © 2021, the author(s), under exclusive license to Springer-Verlag GmbH Germany, part of Springer Nature).

ITA has also been successfully applied to probe aromaticity and conformational effects in porphyrinoid systems [50]. In a study examining two oppositely oriented benzene rings under different spin states (singlet/triplet) and charge states (0 and +2), statistically significant correlations were observed between NICS(1) values and six IT descriptors for four model systems—Singlet 28, Singlet 30, Triplet 28, and Triplet 30 (Table 1). As shown in Figure 4, strong correlations between NICS values and *I_G_* were found for Möbius, Hückel, and twisted conformations, with opposite trends arising from different conformational transformations, underscoring the sensitivity of IT descriptors to electron delocalization patterns.

Beyond static structure, ITA also tracks how electron-density information changes under external perturbations, connecting stability, field response, and reactivity within the same density-based framework.

### 4.4. Stability, External Fields, Electrophilicity, and Nucleophilicity

**Figure 5:**
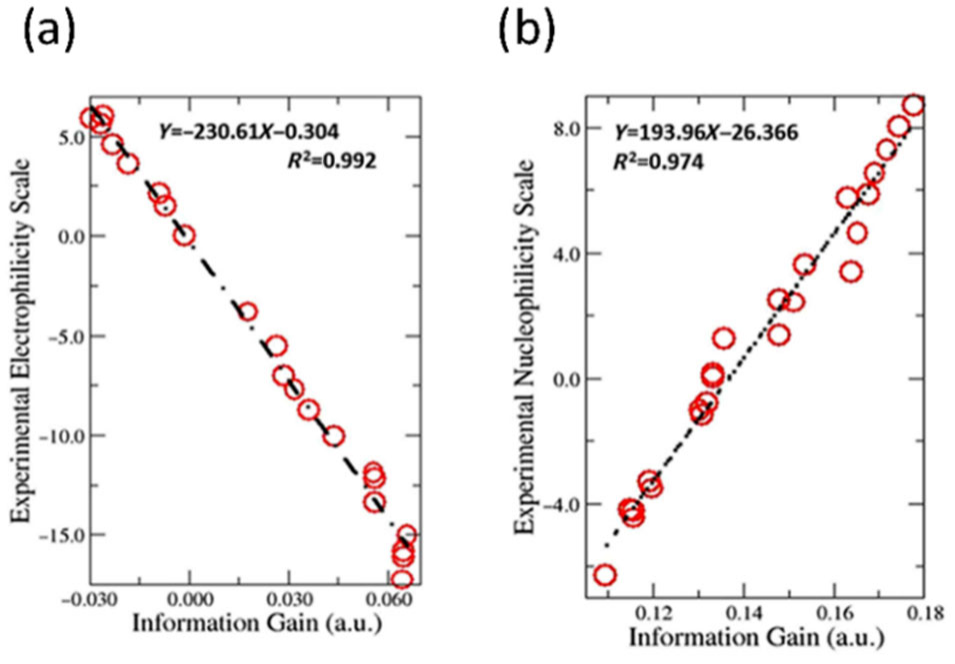
Correlation between experimental (**a**) electrophilicity and (**b**) nucleophilicity scales and the corresponding calculated *I_G_* values. (Reprinted from Reference [49] with permission from John Wiley and Sons. © 2019, Wiley Periodicals, Inc.).

**Scheme 2:**
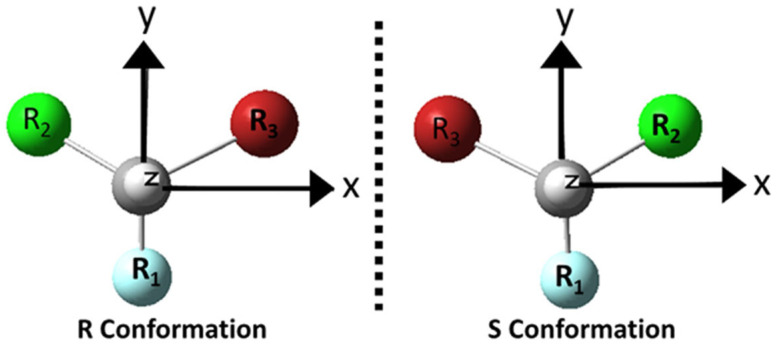
Schematic representation of the studied chiral molecules. The stereogenic center is a carbon atom, with R_1_, R_2_, and R_3_ as substituent groups. The z-axis is aligned with the C–H bond, and an external electric field is applied along the +x direction. A total of 74 molecular pairs were investigated, with R_1_, R_2_, and R_3_ = F, Cl, Br, Me, Et, CN, CF_3_, OH, BH_2_, PH_2_, AsH_2_, SeH, SH, SiH_3_, and Ar. (Reprinted from Reference [47] with permission from Elsevier. © 2020, Elsevier B.V. All rights reserved).

**Figure 6:**
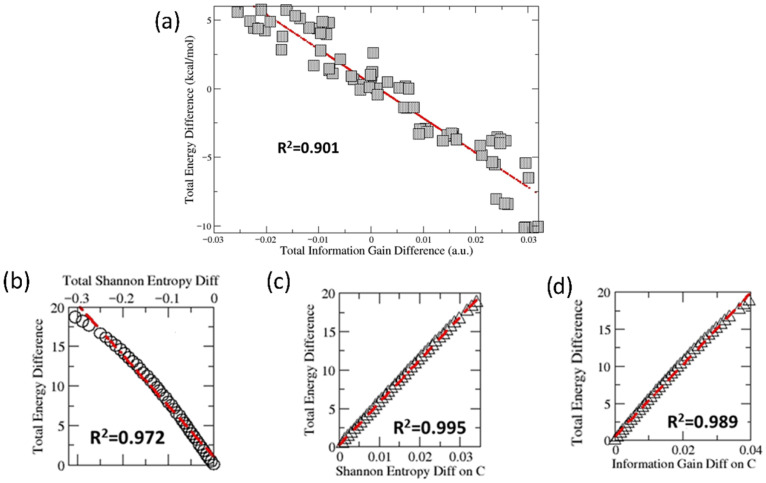
Correlation of the total energy difference (Δ*E*, R–S) with (**a**) total information gain difference (Δ*I_G_*), (**b**) total Shannon entropy difference (Δ*S_S_*), (**c**) Shannon entropy difference at the central carbon, and (**d**) information gain difference at the central carbon. (Reprinted from Reference [47] with permission from Elsevier. © 2020, Elsevier B.V. All rights reserved).

IT quantities have further been employed to analyze stability, electrophilicity, nucleophilicity, and bonding interactions [49]. For example, correlations between experimental electrophilicity and nucleophilicity scales and *I_G_* values are presented in Figure 5a,b. Studies on fullerene isomers (C_44_, C_48_, C_52_, and C_60_) revealed a near-linear relationship between total energy differences and Shannon entropy differences (($∆E=0.254∆S_{S}-0.003,$ *R*^2^ = 0.983) [54]. Additionally, Chen et al. [47] explored the influence of external electric fields on chiral molecules (Scheme 2) using IT descriptors. For 74 molecular pairs, a strong correlation (*R*^2^ = 0.901) was obtained between ∆*E* and Δ*I_G_* under a 0.005 a.u. field applied along the x-axis (Figure 6a). Systematic variation in field strength (0.0–0.02 a.u.) in CHClBrMe demonstrated field-induced structural and electronic rearrangements, with Δ*I_G_* correlating strongly with ∆*E* (Figure 6b–d).

### 4.5. Insights into Chemical Bonding

Beyond reactivity trends, ITA has provided new insights into chemical bonding. Alongside traditional density-based indicators such as the electron localization function and density Laplacian, the Pauli energy has recently been shown to effectively identify strong covalent interactions. A dimensionless strong covalent interaction (SCI) index [55] derived from the Pauli energy reveals characteristic isosurface patterns, viz., dumbbell, torus, and four-lobed shapes corresponding to double, triple, and quadruple bonds, respectively. This methodology has enabled the identification of quintuple and sextuple bonds [55,56] and offered a stringent test for approximate kinetic energy density functionals [57].

### 4.6. Noncovalent Interactions and Cooperativity

**Figure 7:**
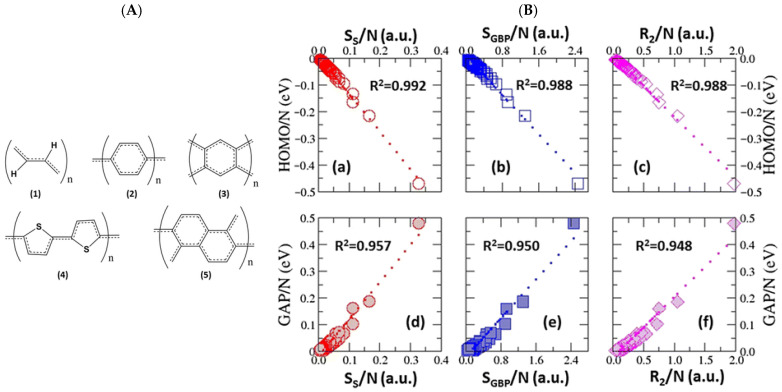
(**A**) Repeating units consisting of (**1**) trans-ethylene, (**2**) benzene, (**3**) naphthalene, (**4**) dithienyl, and (**5**) phenanthrene chains. (**B**) Strong linear correlations between frontier orbital properties and ITA quantities for the systems shown in the left panel. N is the total number of electrons in the polymeric system for all five systems put together. (**a**–**f**) represent the strong linear correlation of information-theoretic quantities with highest occupied molecular orbital (HOMO) and HOMO/lowest unoccupied molecular orbital (LUMO) gap values. R_2_ is the Rényi entropy of order 2. (Reprinted from Reference [61] with permission from Springer. © 2016, Springer-Verlag Berlin Heidelberg).

For systems dominated by noncovalent interactions, ITA has been used to quantify cooperativity effects in atomic and molecular clusters [58,59,60]. Charged systems exhibit negative cooperativity, whereas homogeneous neutral systems display positive cooperativity [60]. From an ITA perspective, these two behaviors arise from fundamentally different electronic origins: electrostatic interactions govern negative cooperativity, while exchange–correlation and steric effects dominate positive cooperativity. Remarkably, IT descriptors have also been shown to capture orbital-related properties. Strong correlations were established between IT quantities and frontier orbital energies, HOMO–LUMO gaps, and oxidation states across multiple polymeric systems (Figure 7) [61].

Thus, whether the problem concerns aromaticity, regioselectivity, or activation barriers, IT descriptors consistently translate chemical behavior into patterns of information flow in the electron density.

### 4.7. Reaction Barriers and Selectivity

**Figure 8:**
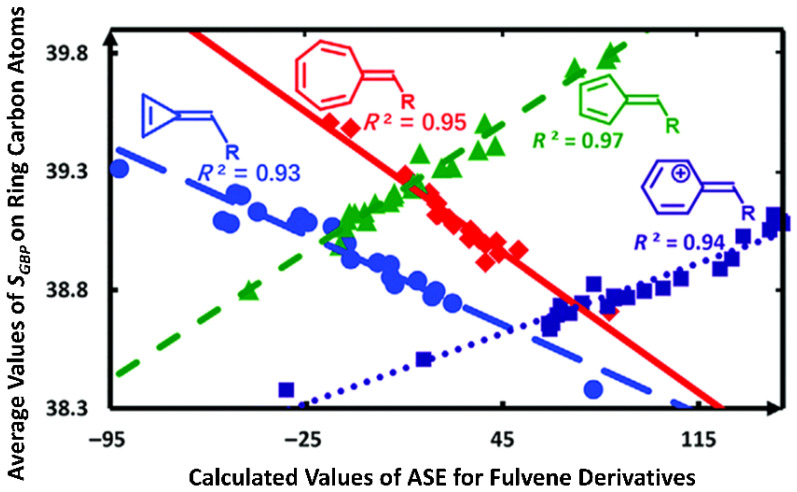
Correlation between the aromaticity index ASE and the ITA quantity $S_{GBP}$ for various substituted fulvene derivatives. (Reprinted from Reference [62] with permission from the Royal Society of Chemistry. © 2017, the Owner Societies).

Finally, ITA has provided fresh perspectives on aromaticity, antiaromaticity, and reaction barrier heights. Using substituted fulvene derivatives, cross-correlations between IT descriptors (e.g., *S_GBP_*) and aromatic stabilization energies (ASE) revealed ring-size-dependent trends consistent with Hückel’s 4n + 2/4n electron rule (Figure 8) [62]. In electrophilic aromatic substitution reactions, Hirshfeld charges and information gain successfully rationalized ortho/para vs. meta directing effects and accurately predicted activation barriers, with correlation coefficients exceeding 0.96 [63]. Similarly, in S_N_2 reactions, barrier heights were shown to depend not only on electrostatic factors but also on steric and information-theoretic contributions, with multiple IT descriptors jointly providing reliable predictions [64,65].

## 5. ITA in QSAR/QSPR and Toxicity Prediction

In this context, QSAR/QSPR modeling represents a natural extension of the same principle: macroscopic properties are learned as functions of how molecular electron-density information is organized and redistributed.

QSAR/QSPR modeling plays a central role in predicting physicochemical and toxicity-related properties of chemical systems, particularly when experimental measurements are scarce, costly, or hazardous. In this context, IT descriptors derived from the electron density offer physically meaningful and computationally efficient variables for modeling structure–property relationships. Their integration with conceptual DFT provides a robust and interpretable framework for predicting environmentally relevant properties that are closely linked to toxicity and bioaccumulation.

**Scheme 3:**
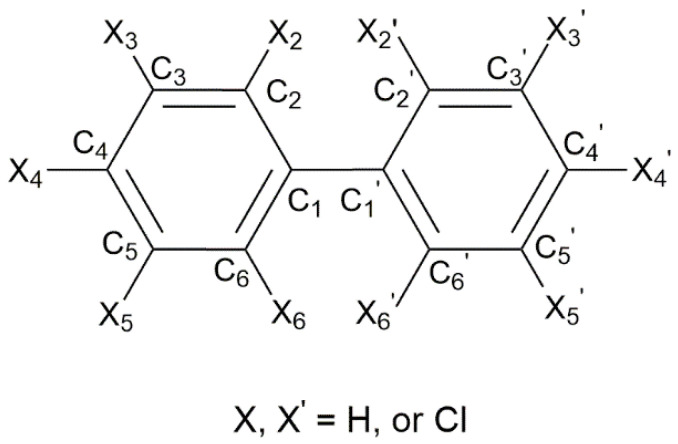
Template of the biphenyl molecule with atom numbering. (Reprinted from Reference [29] with permission from Springer Nature. © 2023, the author(s), under exclusive licence to Springer Nature Switzerland AG).

A recent application of this integrated framework focused on the prediction of the logarithm of the octanol–water partition coefficient (log *K*_OW_) for 133 polychlorinated biphenyls (PCBs, Scheme 3), a class of persistent organic pollutants of significant environmental concern [29]. In this study, experimental log *K*_OW_ values were used as the dependent variable, while electrophilicity (*ω*), its square (*ω*^2^), *S_S_*, and *S_GBP_* served as independent variables. The linear regression model combining *ω* with IT descriptors yielded a high coefficient of determination (*R*^2^ = 0.926). The statistical parameters of the regression models are summarized in Table 2, and the low Root-Mean-Square Error values indicate the robustness and reliability of the selected descriptors. Internal validation using the leave-one-out cross-validation (LOO-CV) method further confirmed the predictive strength of the model, yielding a cross-validated coefficient $R_{CV}^{2}$ = 0.9208.

The methodology was subsequently extended to simultaneously predict log *K*_OW_ and the enthalpy of vaporization (∆_vap_*H*_m_) for PCBs by exploring different combinations of CDFT and IT descriptors [30]. Descriptor selection guided by Pearson correlation coefficient analysis revealed strong interdependencies among several conceptual and IT quantities. For log *K*_OW_ prediction, a regression model incorporating *I_G_*, *g*_1_, *g*_2_, electron affinity (*EA*), and chemical hardness (*η*) achieved an *R*^2^ value of 0.9342 and is expressed as

Calc (log *K*_OW_) = −0.899 × *I_G_* + 0.106 × *g*_1_ − 0.135 × *g*_2_ + 61.640 × *EA +* 15.120 × *η* − 7.309

**Figure 9:**
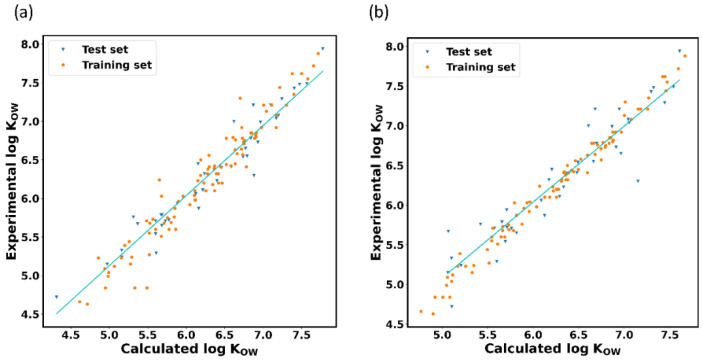
Plots comparing calculated and experimental log *K*_OW_ values for training and test set molecules using *I_G_*, *g*_1_, *g*_2_, *EA*, and *η* descriptors in (**a**) MLR analysis and (**b**) RF calculations. (Reprinted from Reference [30] with permission from Springer Nature. © 2024, Indian Academy of Sciences).

The dataset was divided into a training set of 93 PCBs and a test set of 40 PCBs. Using 70% of the dataset for model training, the refined regression equation yielded an *R*^2^ value of 0.9362 for the training set and 0.9228 for the test set ($R_{adj}^{2}\;$= 0.9115, SD = 0.2097). Validation using the Random Forest (RF) method resulted in *R*^2^ = 0.8807 ($R_{adj}^{2}$ = 0.8632, SD = 0.2607) for the test set. A comparison between calculated and experimental log *K*_OW_ values obtained from both regression and RF models is presented in Figure 9. For the prediction of ∆_vap_*H*_m_, the best-performing regression model achieved an R^2^ value of 0.8662 and is given by

Calc (∆_vap_*H*_m_) = −28.766 × *I_F_* + 0.478 × *g*_3_ + 2.276 × *η* − 239.692 × *S_S_* + 571.826 × *S_GBP_* − 199,240.844

These results demonstrate that hybrid CDFT–ITA models can accurately predict both partitioning behavior and thermodynamic properties relevant to environmental fate and toxicity. Overall, this section highlights the effectiveness of IT descriptors in QSAR/QSPR modeling and underscores their potential for predictive toxicology and environmental risk assessment.

## 6. Synergy Between IT Descriptors and Conceptual DFT

The synergistic integration of IT descriptors with conceptual density functional theory has emerged as a powerful strategy for achieving a more comprehensive and physically transparent description of chemical reactivity. While CDFT provides well-established global and local descriptors rooted in energy derivatives, IT quantities capture complementary aspects of electron density distribution, localization, and delocalization. Together, these two frameworks offer enhanced predictive capability and deeper chemical insight, particularly when combined with modern data-driven and machine-learning approaches.

Periodic trends based on CDFT and IT descriptors have been systematically investigated for elements ranging from hydrogen to barium (Z = 1–56) [31]. Traditional global descriptors such as ionization potential (*IP*), *EA*, electronegativity (*χ*), and *η* exhibit periodic behavior consistent with the Aufbau principle and standard electronic structure concepts. Interestingly, *ω* displays a distinct pattern, with noble gases showing the lowest *ω* values within each period, reflecting their minimal reactivity and stable closed-shell configurations. Similar periodic trends are observed for electro-accepting and electro-donating powers. IT quantities, including Shannon entropy, GBP entropy, and the Shannon entropy of the shape function, also exhibit pronounced local minima for noble gas elements, reinforcing their unique electronic character.

**Figure 10:**
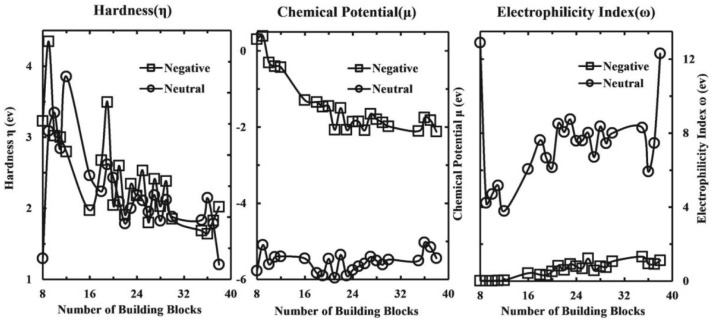
Variation in hardness, chemical potential, and electrophilicity index in boron clusters; squares correspond to negatively charged species, and circles to neutral species. (Reprinted from Reference [34] with permission from Taylor & Francis. © 2022, Informa UK Limited, trading as Taylor & Francis Group).

The combined CDFT–ITA framework has also been applied to analyze cooperativity and reactivity in neutral and anionic boron clusters ($B_{n}$/$B_{n}^{-}$, *n* = 8–38) [34]. For neutral clusters, exchange–correlation and electrostatic interactions contribute comparably to cooperativity, with minimal steric effects. In contrast, anionic clusters exhibit multifaceted negative cooperativity, with total energy variations not dominated by any single energetic component. Global descriptors such as *η*, chemical potential (*µ*), and *ω* display systematic differences between neutral and charged clusters, as illustrated in Figure 10. While most clusters show similar hardness values, anionic species generally possess higher chemical potentials and lower electrophilicity indices. IT descriptors further elucidate the origin of cooperativity in these systems. For neutral clusters, *Sₛ*, *I_F_*, and *S_GBP_* exhibit strong correlations with interaction energy per building block (*Eₙ*), as summarized in Table 3. In contrast, for anionic clusters, only the second-order Rényi entropy (*R_2_*) shows a strong positive correlation, while relative Rényi entropy ($R_{2}^{r}$) contributes negatively. Local reactivity analyses using density- and information-based Fukui functions reveal that electrophilic and nucleophilic regions are predominantly located at the cluster edges. In $B_{23}^{-}$, electrophilic regions are concentrated near the poles, whereas nucleophilic regions are distributed along the periphery. In $B_{36}^{-}$, partial overlap of these regions suggests dual reactivity for certain atoms, a feature further confirmed by dual descriptors.

**Scheme 4:**
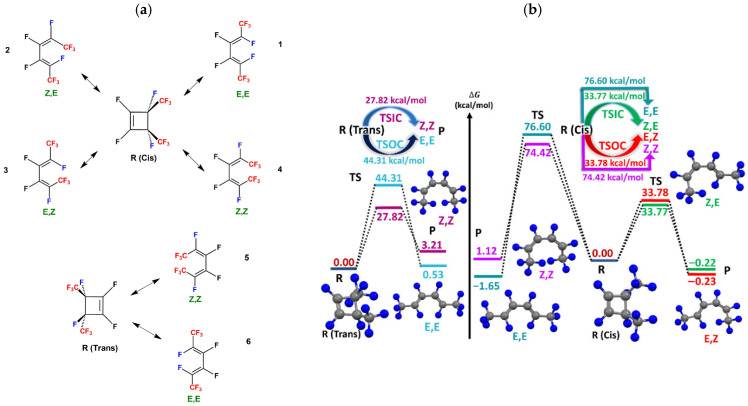
(**a**) Schematic representation of Thermal ring-opening reaction of cis-and trans-perfluoro-3,4-dimethylcyclobutene systems. The numbering corresponding to the studied reactions has been included. (**b**) Reaction energy profiles of ring-opening cis-and trans-perfluoro-3,4-dimethylcyclobutene systems. (TSIC/TSOC denotes Transition State Inward/Outward Conrotatory, R = Reactant, P = Product.) (Reprinted from Reference [13] with permission from the Royal Society of Chemistry. © 2025, the Owner Societies).

**Figure 11:**
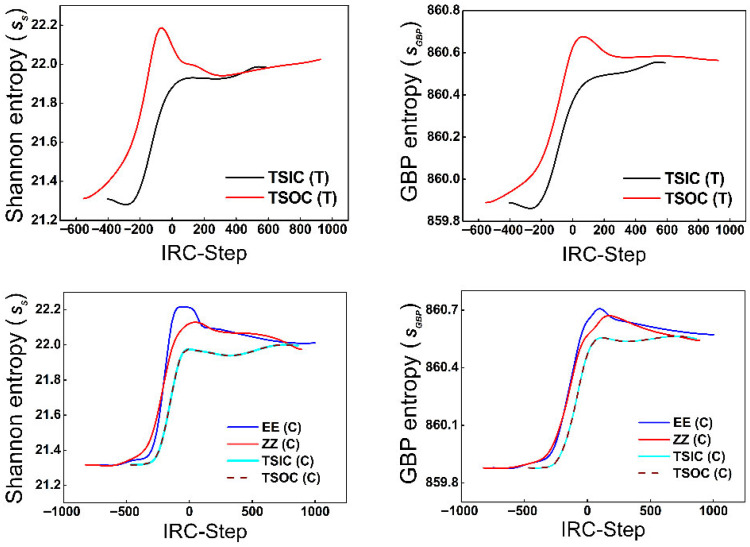
Variation in information-theoretic descriptors along the IRC for cis- and trans-perfluoro-3,4-dimethylcyclobutene systems. (Reprinted from Reference [13] with permission from the Royal Society of Chemistry. © 2025, the Owner Societies).

A recent density-based investigation combined conceptual DFT and IT descriptors to elucidate the origin of unusual torquoselectivity in the electrocyclic ring-opening of perfluoro-3,4-dimethylcyclobutene (Scheme 4) [13]. Global and local CDFT descriptors, particularly electrophilicity and softness, were systematically correlated along the intrinsic reaction coordinate using multiple linear regression analysis. The results revealed exceptionally strong correlations between global electrophilicity and the local electrophilicities of the carbon atoms directly involved in *σ*-bond cleavage, demonstrating that bond rupture governs the evolution of global reactivity along the reaction pathway. Extension of the analysis to all ring carbons further improved the correlations, highlighting the role of *π*-bond formation in the product region. IT descriptors provided complementary insight into reaction selectivity: *S_S_* and *S_GBP_* identified lower electronic uncertainty and more directed density reorganization along the kinetically favored inward conrotatory pathway (Figure 11). The combined CDFT–ITA framework thus offers a density-based explanation for stereochemical outcomes that are not adequately captured by traditional orbital-based models, establishing a predictive route for understanding torquoselectivity in electrocyclic reactions.

**Figure 12:**
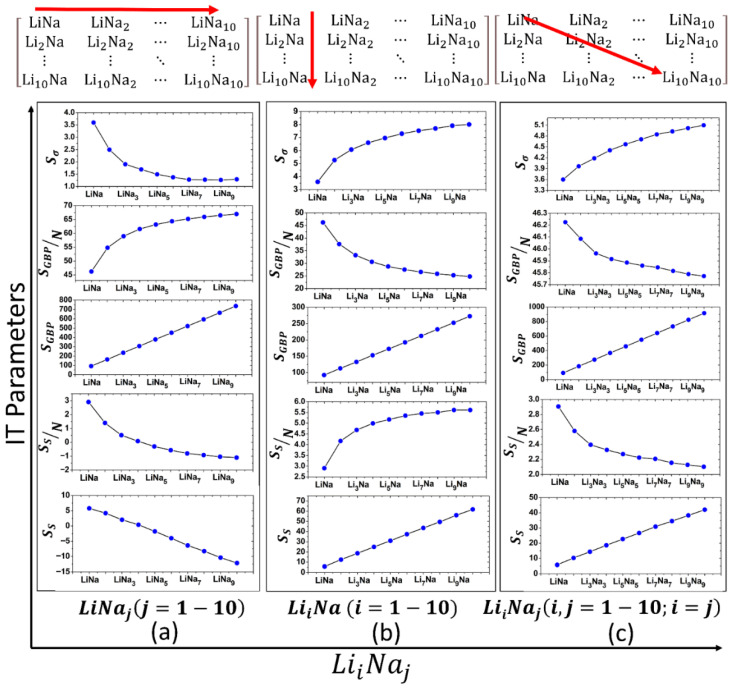
Calculated information-theoretic parameters for Li_i_Na_j_ clusters with varying values of i and j. (Reprinted from Reference [66] with permission from Chemistry Europe. © 2026, Wiley-VCH GmbH).

The synergy between conceptual DFT and the information-theoretic approach has also been demonstrated in the study of monometallic and bimetallic Li–Na nanoalloy clusters [66]. CDFT descriptors such as *IP*, *η*, *χ*, and *ω* exhibit pronounced odd–even oscillations with cluster size and composition, reflecting quantum size effects and electron shell closure. These trends are consistent with the maximum hardness and minimum electrophilicity principles, particularly for sodium-rich clusters. Complementary ITA descriptors, including *S_S_* and *S_GBP_*, provide a nuanced picture of electron delocalization in both real and phase space (Figure 12). While Shannon entropy captures changes in spatial density distribution, GBP entropy reflects kinetic and phase-space complexity, leading to composition-dependent and sometimes opposing trends. Na-rich clusters show enhanced delocalization in energy space, whereas Li-rich clusters display increased spatial delocalization, in agreement with CDFT-predicted variations in hardness, polarizability, and electrophilicity. Together, the CDFT–ITA analysis establishes a coherent density-based interpretation of how alloying and size modulate stability, reactivity, and electronic structure in alkali-metal nanoalloys.

**Scheme 5:**
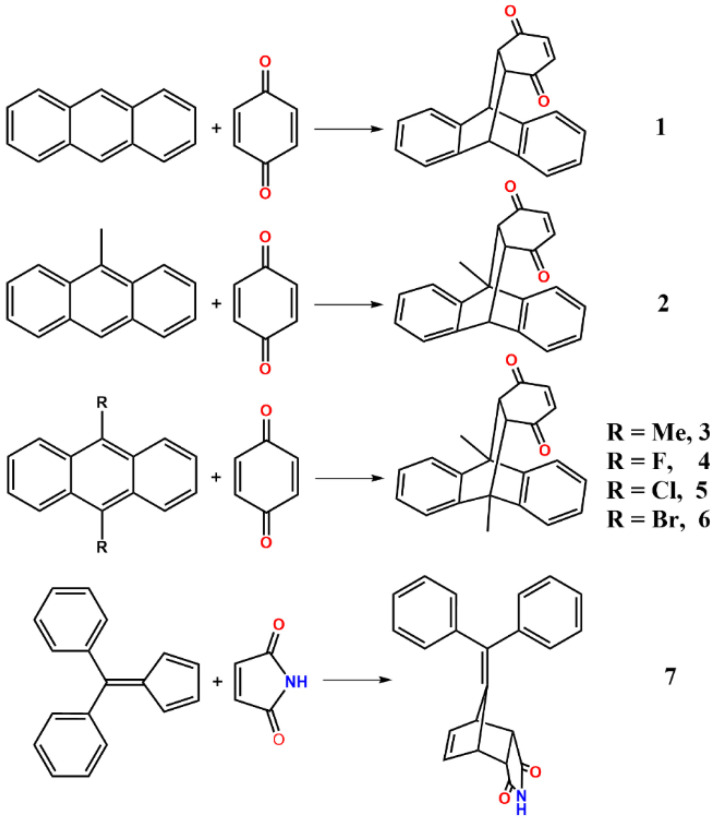
The selected mechanochemical Diels-Alder reactions (**1**) anthracene + p-benzoquinone, (**2**) 9-methylanthracene + p-benzoquinone, (**3**) 9,10-dimethylanthracene + p-benzoquinone, (**4**) 9,10-difluoroanthracene + p-benzoquinone, (**5**) 9,10-dichloroanthracene + p-benzoquinone, (**6**) 9,10-dibromoanthracene + p-benzoquinone, and (**7**) diphenylfulvene + maleimide. (Reprinted from Reference [14] with permission from Chemistry Europe. ©2025 Wiley-VCH GmbH).

The combined application of IT descriptors and conceptual DFT has recently been extended to mechanochemical retro Diels–Alder reactions (Scheme 5) to elucidate force-induced reactivity under external mechanical stress [14]. IT quantities such as *S_S_* and *S_GBP_* were analyzed as first-order responses to the applied force, revealing systematic changes in electronic uncertainty and density redistribution near the bond-rupture region. These descriptors capture the progressive localization or delocalization of electron density as the reacting fragments are mechanically driven toward dissociation, offering a density-based interpretation of mechanochemical activation. Notably, ITA measures exhibit distinct trends for the diene and dienophile fragments, reflecting their different electronic responses to force and complementing the behavior of global and local CDFT descriptors. The correlation between IT parameters with the variation in applied external force demonstrate the ability of ITA to track mechanochemical pathways and distinguish substituent effects on reaction feasibility. Overall, this study highlights IT descriptors as sensitive and physically meaningful probes for understanding force-modulated chemical reactivity beyond conventional energy-based analyses.

Overall, the synergy between CDFT and ITA augmented by machine-learning validation provides a unified and versatile framework for understanding chemical reactivity across atoms, molecules, and clusters. This integrated approach not only enhances predictive accuracy but also deepens conceptual understanding, highlighting the complementary nature of energy-based and density-based descriptions of electronic structure. Together, these examples demonstrate that ITA is not a collection of isolated descriptors, but a unified density-based language for chemical structure and reactivity, applicable across scales, bonding regimes, and external perturbations.

## 7. Challenges, Limitations, and Future Directions

Despite the considerable progress achieved by ITA and CDFT in rationalizing chemical structure and reactivity, several conceptual and practical challenges remain that must be addressed to advance toward a comprehensive density-based reactivity theory.

One of the major strengths of ITA lies in its ability to establish statistically robust correlations between density-based descriptors and experimentally measurable properties across diverse chemical systems. Strong linear and multilinear relationships between IT measures and energetic quantities, acidity scales, electrophilicity, and reactivity trends have repeatedly demonstrated the predictive power of IT descriptors. Nevertheless, these correlations are often system-dependent, and their transferability across chemically heterogeneous datasets remains limited. Different classes of molecules frequently require separate scaling relationships, indicating that a universal information-based reactivity scale has yet to be realized.

Another important limitation concerns sensitivity to the underlying electronic structure method. Although IT descriptors are explicit density functionals, their numerical values can still be influenced by the choice of exchange–correlation functional, basis set, and population partitioning scheme. This sensitivity becomes particularly pronounced for large systems, charged species, and weakly bound complexes, where subtle density redistributions play a critical role. Moreover, while ITA offers an orbital-free framework, the interpretation of certain descriptors especially local quantities can be less intuitive without complementary chemical context.

From a conceptual standpoint, reactivity represents a system’s intrinsic tendency to undergo transformation whether through bond rearrangement, electron or proton transfer, conformational change, or response to external perturbations. Traditional molecular orbital theory provides intuitive but inherently non-observable constructs, whereas DFT asserts that the ground-state electron density alone determines all properties. The challenge, therefore, is to determine whether different aspects of reactivity can be uniquely and systematically mapped onto well-defined density functionals, or whether multiple descriptors are required to capture distinct reactive tendencies.

Encouraging progress in this direction has been made by employing information-theoretic quantities such as information gain, relative entropy, and kinetic energy-based measures to describe steric effects, electrophilicity, nucleophilicity, and regioselectivity. Notably, the interpretation of electrophilic aromatic substitution in terms of information-based descriptors has provided a density-centric explanation of ortho/para versus meta directing effects, bypassing orbital arguments altogether. However, extending such successes to stereoselectivity, enantioselectivity, and complex catalytic environments remains an open frontier.

Another limitation arises in the treatment of large molecular systems and reaction networks. The computational cost associated with high-quality density evaluations, combined with the increasing dimensionality of descriptor spaces, can hinder the practical deployment of ITA for biomolecules, materials, or multistep reaction mechanisms. In this context, emerging data-driven approaches offer a promising route forward. Machine-learning models trained on ITA and CDFT descriptors can capture nonlinear relationships, improve generalization across chemical space, and dramatically reduce computational expense by learning surrogate models for expensive quantum-chemical calculations. Feature-selection strategies further enhance interpretability by identifying the most chemically meaningful descriptors.

Looking ahead, several theoretical avenues deserve particular attention. These include systematic comparisons between different relative entropy measures, such as Kullback–Leibler divergence and relative Rényi entropy; deeper exploration of local information-theoretic quantities and their role in governing selectivity; and the construction of new density functionals tailored to specific reactivity phenomena. Equally important is the development of unified, physically motivated scales for acidity/basicity and electrophilicity/nucleophilicity that remain valid across chemically diverse systems.

Unlike earlier surveys that largely catalog individual descriptors, this review emphasizes the conceptual unification of ITA, CDFT, DFRT, and emerging machine-learning strategies, highlighting their collective role in advancing a fully density-based theory of chemical reactivity.

The wide range of applications surveyed in this review from bonding analysis and aromaticity to regioselectivity, toxicity, and materials screening are united by a common conceptual foundation: all are expressions of how chemical behavior emerges from the information content and redistribution of the electron density. In this sense, ITA does not replace orbital-based models with another set of descriptors but reframes chemical reactivity itself as a density-driven information flow problem.

In summary, while ITA has already demonstrated its ability to quantify molecular structure and reactivity using simple density functionals, significant challenges remain in universality, transferability, and interpretability. Addressing these issues through theoretical refinement, methodological standardization, and integration with data-driven techniques will be crucial for realizing the long-term goal of a fully density-based chemical reactivity theory. The continued convergence of information theory and DFT thus represents not a conclusion, but the opening of a broad and fertile research landscape.

## Figures and Tables

**Table 1:** Correlation coefficients (R) between NICS(1) values and IT quantities, using the Möbius conformation as the reference. (Reprinted from Reference [50] with permission from Springer Nature. © 2021, the author(s), under exclusive licence to Springer-Verlag GmbH Germany, part of Springer Nature).

	Singlet 28		Singlet 30		Triplet 28		Triplet 30	
	M-H-M	M-T-M	M-H-M	M-T-M	M-H-M	M-T-M	M-H-M	M-T-M
$I_{F}$	0.918	−0.798	−0.983	0.929	−0.965	0.946	0.980	−0.417
$R_{2}^{r}$	0.961	−0.997	−0.977	0.919	−0.923	0.963	0.994	−0.792
$R_{3}^{r}$	0.965	−0.997	−0.981	0.924	−0.921	0.965	0.993	−0.823
$I_{G\;}$	0.958	−0.996	−0.976	0.919	−0.935	0.963	0.994	−0.780
*g*_1_	−0.994	0.991	0.942	−0.887	0.922	−0.951	−0.953	0.967
*g*_2_	0.983	−0.965	−0.972	0.927	−0.962	0.964	0.948	−0.846

**Table 2:** Statistical parameters and corresponding regression equations for the full dataset comprising 133 PCB molecules. (Reprinted from Reference [29] with permission from Springer Nature. © 2023, the author(s), under exclusive licence to Springer Nature Switzerland AG).

Regression Equations:	*R* ^2^	*R*_adj_^2^	Root-MSE (SD)	*R*
log *K*_OW_ = −1.49766 + 184.05245 × *ω*	0.8460	0.8448	0.2744	0.9198
log *K*_OW_ = 2.43437 + 2136.30594 × *ω*^2^	0.8313	0.8300	0.2847	0.9117
log *K*_OW_ = 6.57862 − 0.02767 × *S_S_*	0.8413	0.8401	0.2778	0.9172
log *K*_OW_ = 2.13932 + 0.00393 × *S_GBP_*	0.8419	0.8407	0.2777	0.9175
log *K*_OW_ = −7.35828 + 461.34759 × *ω* − 3253.98814 × *ω*^2^	0.8544	0.8532	0.2682	0.9243
log *K*_OW_ = 2.12635 + 102.15868 × *ω* − 0.01496 × *S_S_*	0.9244	0.9238	0.2010	0.9615
log *K*_OW_ = −0.2693 + 101.97723 ×*ω* + 0.00213 × *S_GBP_*	0.9245	0.9240	0.2010	0.9615
log *K*_OW_ = 4.38689 + 1146.61427 ×*ω*^2^ − 0.01565 × *S_S_*	0.9220	0.9214	0.2038	0.9602
log *K*_OW_ = 1.87643 + 1144.56208 ×*ω*^2^ + 0.00222 × *S_GBP_*	0.9222	0.9216	0.2035	0.9603
log *K*_OW_ = −171.10256 + 1.08017 × *S_S_* + 0.15713 × *S_GBP_*	0.8530	0.8519	0.2693	0.9236
log *K*_OW_ = 0.59242 + 172.9107 × *ω* − 810.66909 × *ω*^2^ − 0.01466 × *S_S_*	0.9249	0.9243	0.2004	0.9617
log *K*_OW_ = −1.71257 + 170.7434 × *ω* − 787.73512 × *ω*^2^ + 0.00208 × *S_GBP_*	0.9250	0.9244	0.2001	0.9618
log *K*_OW_ = −67.1329 + 98.61511 × *ω* + 0.41739 × *S_S_* + 0.06138 × *S_GBP_*	0.9261	0.9255	0.1987	0.9623
log *K*_OW_ = −87.58681 + 1103.53735 × *ω*^2^ + 0.55787 × *S_S_* + 0.08141 × *S_GBP_*	0.9251	0.9245	0.2003	0.9618
log *K*_OW_ = −63.37192 + 117.72111 × *ω* − 216.46954 × *ω*^2^ + 0.39144 × *S_S_* + 0.05769 × *S_GBP_*	0.9261	0.9256	0.1989	0.9624

**Table 3:** The correlation coefficient (*R*) between the interaction energy per building block (*E_n_*) and IT descriptors for both neutral and anionic boron clusters. (Reprinted from Reference [34] with permission from Taylor & Francis. © 2022, Informa UK Limited, trading as Taylor & Francis Group).

R	Negative	Neutral
${{(S}_{S})}_{n}$	−0.599	0.987
${{(I}_{F})}_{n}$	−0.372	0.958
${{(S}_{GBP})}_{n}$	−0.841	0.989
${{(I}_{G})}_{n}$	0.659	−0.104
${{(R}_{2})}_{n}$	0.924	−0.982
${\left(R_{2}^{r}\right)}_{n}$	0.904	−0.982

## Data Availability

No new data were created or analyzed in this study. Data sharing is not applicable to this article.
